# VEGFR2 Expression Is Differently Modulated by Parity and Nulliparity in Mouse Ovary

**DOI:** 10.1155/2018/6319414

**Published:** 2018-09-16

**Authors:** Valentina Di Nisio, Gianna Rossi, Roberto Iorio, Cristina Pellegrini, Guido Macchiarelli, Gian Mario Tiboni, Sabrina Petricca, Sandra Cecconi

**Affiliations:** ^1^Department of Life, Health and Environmental Sciences, University of L'Aquila, 67100 L'Aquila, Italy; ^2^Department of Biotechnological and Applied Clinical Science, University of L'Aquila, 67100 L'Aquila, Italy; ^3^Department of Medicine and Aging Science, University “G. D'Annunzio”, Chieti-Pescara, Chieti, Italy

## Abstract

Parity and nulliparity exert opposite effects on women's health, as parity is considered a protective factor for several reproductive diseases. This study is aimed at determining if ovarian VEGF and VEGFR2 expression are differently modulated in the ovaries of parous and nulliparous mice. To this end primiparous and nulliparous fertile mice were sacrificed at postovulatory stage. Whole ovaries, corpus luteum, and residual stromal tissues were analyzed to assess VEGF/VEGFR2 expression levels. Ovarian mRNA amounts of* Vegfa* (*120* and* 164*) and* Vegfr2* were comparable between primiparous and nulliparous mice; both isoforms and receptor were accumulated mainly in corpus luteum tissues. VEGF 120 and 164 protein accumulation and distribution mirrored that of mRNA. Conversely, VEGFR2 protein content was significantly higher in ovaries of nulliparous mice and was more efficiently phosphorylated in ovaries of primiparous mice. In both groups, VEGFR2 was preferentially expressed in corpus luteum, while its phosphorylated form was equally distributed in two somatic compartments. We suggest that parity influences VEGFR2/phospho-VEGFR2 expression and tissue distribution. This difference could be part of a more complex mechanism that at least in mice is activated after the first pregnancy and likely aims to preserve female health.

## 1. Introduction

A significant decline of fertility rate is occurring in developed countries, mainly due to economic problems and lifestyle choices. Therefore, many young women postpone pregnancy at older ages, often ignoring that quantity and quality of oocytes both decrease from 35 years onward [1, 2]. In addition, young women can experience fertility problems because of several pathological conditions affecting the ovary, first of all cancer [3–5], thereby increasing the number of childless women.

A key question is whether infertility and, even more, nulliparity can be considered as risk factors for ovarian cancer (OC) onset [3, 6, 7]. As a matter of fact, the risk seems to be increased also in women affected by polycystic ovarian syndrome (PCOS) [8], corpus luteum insufficiency [9], and endometriosis [10]. In women undergoing stimulation protocols during IVF procedures the question is still debated [11, 12]. Interestingly, all these diseases are characterized at the molecular level by elevated hypoxia and overexpression of proangiogenic factors, especially vascular endothelial growth factor (VEGF) and vascular endothelial growth factor receptor type 2 (VEGFR2) [8, 13–15]. This is not surprising, as in the adult mammalian ovary a delicate balancing of pro/antiangiogenic factors participates in the physiological modulation of cyclical angiogenesis and vascular regression [8, 16–19].

To date, many efforts aimed at demonstrating the link between female infertility and increased OC risk have led to unconclusive results. It is noteworthy that, in mice, a full-term pregnancy significantly reduces the risk of developing chemically induced mammary cancers, compared with nulliparous animals [20, 21]. In women, a full-term pregnancy seems to reduce the risk of developing different reproductive cancers, such as breast [22], ovarian [23], and endometrial cancer [24], compared with nulliparity [6, 25]. However, such a protective effect seems to be exerted only when the first full-term pregnancy occurs before 30 years of age [26].

The present study explored how pregnancy can exert its protective effects by assessing if VEGF and VEGFR2 expression were differently modulated in the ovaries of adult primiparous (mothers, M), compared with nulliparous (virgins, V) mice.

## 2. Materials and Methods

### 2.1. Chemicals

The chemicals used were purchased from the following sources: rabbit polyclonal VEGFA (sc-507) and phospho-ERK1/2 (Thr202/Tyr204; sc-16982-R); mouse monoclonal Flk1 (sc-6251; VEGFR2), ERK1/2 (sc-135900), and *α*/*β* tubulin (sc-51502); goat anti-rabbit IgG conjugated to horseradish peroxidase (HRP) (sc-2004) and goat anti-mouse IgG conjugated to HRP (sc-2005) from Santa Cruz Biotechnology (Santa Cruz, CA, USA). Rabbit monoclonal phospho-VEGFR-2 (pY1173; #2478), rabbit polyclonal phospho-PLC*γ*1 (Tyr783; #2821), and PLC*γ*1 (#2822) were purchased from Cell Signaling Technology (Beverly, MA, USA). SuperSignal West Pico Chemiluminescent substrate (34080) and RNA*later*™ Stabilization Solution (AM7020) were purchased from Thermo Scientific (Rockford, IL, USA); RNeasy Mini Kit was purchased from Qiagen (Chatsworth, CA); ThermoScript™ RT-PCR Transcription kit was purchased from Invitrogen (Milan, Italy); PowerUp SYBR™ Green Master Mix was purchased from Life Technologies (Carlsbad, CA, USA). Primers were purchased from Integrated DNA Technologies (IDT, Coralville, IA, USA). Eosin–Floxin alcoholic solution (05-10020/L) and Carazzi's Hematoxylin–Nuclear staining (05-06012/L) were purchased from Bio-Optica (Bio-Optica Milano SpA, Milan, Italy). All other reagents were obtained from Sigma-Aldrich Company (St. Louis, MO, USA).

### 2.2. Animals and Ethical Approval

*Mus Musculus *Swiss CD1 female mice (Harlan Italy, Udine, Italy) were housed in an animal facility under controlled temperature (21±1°C) and light (12 h light/day) conditions, with free access to food and water. Mice of the same age (2 months old,* n *= 40) were sorted into 2 groups: (a) mothers (M,* n *= 20) that were mated with males of proven fertility and (b) virgins (V,* n* = 20), unmated, that were kept alone. Once the pregnancy was established, the group of M mice were kept alone until the birth of offspring and weaning. Afterwards, M and V mice (4 months old) were sacrificed at the postovulatory stage of estrus, determined by the analysis of vaginal smears [27]. After collection, ovaries were snap frozen and stored at −80°C for western blotting and histological analysis or for quantitative real-time PCR (qrt-pcr) in RNA*later *Stabilization Solution. When utilized for molecular analyses, ovaries were processed using a rotor-stator tissue homogenizer (Precellys 24, Bertin Technologies) for two cycles of 10 s at 5000 x g.

All experimental procedures involving animals and their care were performed in conformity with national and international laws and policies (European Economic Community Council Directive 86/609, OJ 358, 1 Dec 12, 1987; Italian Legislative Decree 116/92, Gazzetta Ufficiale della Repubblica Italiana n. 40, Feb 18, 1992; National Institutes of Health Guide for the Care and Use of Laboratory Animals, NIH publication no. 85-23, 1985). The project was approved by the Italian Ministry of Health and the internal Committee of the University of L'Aquila. All efforts were made to minimize suffering.

### 2.3. Somatic Cells Retrieval

Corpus luteum tissues (CLT) were obtained as described by Park et al. [28]. Briefly, CLT were dissected from ovaries under light field stereoscope and separated from the residual stromal tissues (RST). Samples were immediately snap frozen at −80°C for western blotting or stored at −80°C for RT-PCR in RNA*later* Stabilization Solution.

### 2.4. H&E Staining

Ovaries were processed and stained according to the protocol of Park et al. [28]. After fixation overnight (o.n.) in 4% formalin, ovaries were embedded in paraffin, sectioned (5 *μ*m/section), stained, and mounted. Sections were examined using StereoZoom® Leica S8 APO and images were acquired with Leica EC3 camera.

### 2.5. RNA Isolation and Relative Real-Time PCR

Total RNA was extracted from each sample using the RNeasy Mini Kit, according to manufacturers' protocols. Quality and quantity of extracted RNA were measured by calculation of the optical density with an ND-1000 Spectrophotometer (NanoDrop, Wilmington, DE).

In order to detect mRNA expression levels of genes of interest, qrt-pcr was performed. Briefly, using mRNA as template, single-stranded cDNAs were generated from 500 ng of total RNA by the ThermoScript™ RT-PCR Transcription kit (Invitrogen, Milan, Italy) according to manufacturer's directions.* Vegf 120*,* Vegf 164,* and* Vegfr2* levels were measured by qrt-pcr on the 7500 Fast real-time PCR system with SYBR® Green Technology using 100 ng cDNA mixed to 20 *μ*l of PowerUp SYBR® Green Master Mix (Life technologies) and 500 nM of each forward and reverse primer (Table 1). The thermal cycling conditions were as follows: 2 min at 50°C and 2 min at 95°C, followed by 40 cycles of 95°C for 15 s and 60°C for 60 s. Levels of gene expression were reported as relative units with respect to mRNA levels of Actin gene used as reference gene to normalize each sample, as previously published [29, 30]. Each gene was analyzed in triplicate. Relative quantitative evaluation of mRNAs was performed by comparative ΔΔCt method.

### 2.6. Western Blotting

Samples were resuspended in lysis buffer (50 mM Tris, pH 7.4, 150 mM NaCl, 1 mM EDTA, and 1% Igepal) containing protease inhibitors (1 mM phenylmethylsulphonylfluoride, 1 *μ*g/ml leupeptin, and 1 *μ*g/ml aprotinin) and phosphatase inhibitors (1 mM sodium fluoride, 10 mM sodium pyrophosphate, and 1 mM sodium orthovanadate), homogenized and centrifuged. Protein concentration was determined by Bio-Rad Protein Assay. Sixty *μ*g of protein/sample was loaded onto 8% or 12% gels under reducing conditions, except for the VEGFR2 examined in nonreducing condition. After transfer, blots were incubated with anti-VEGFA (1:200), anti-pERK (1:200), anti-ERK (1:200), anti-pPLC*γ*1 (1:1000), anti-PLC*γ*1 (1:1000), anti-VEGFR2 (1:200), and anti-pVEGFR2 (1:1000) antibodies o.n. at 4°C.

HRP-conjugated goat anti-rabbit IgG (1:5000) and goat anti-mouse IgG (1:5000) were used as secondary antibody (1h, room temperature), and peroxidase activity was detected using a SuperSignal West Pico Chemiluminescent substrate. The nitrocellulose membranes were examined using the Alliance LD2-77WL imaging system (Uvitec, Cambridge, UK). Densitometric quantification was performed with the public-domain software NIH Image V.1.62 and standardized using tubulin as loading control. pVEGFR2, pERK, and pPLC*γ*1 signals were normalized to the respective total of VEGFR2, ERK, and PLC*γ*1, as previously described [31].

### 2.7. Statistical Analysis

All experiments were performed at least three times, and data were expressed as mean percentage ± SEM. Data from V mice were compared to data obtained from M mice, which were arbitrarily set as 100%. Also data from RST were compared to CLT, which were arbitrarily set as 100%. Experimental results of molecular analysis were analyzed using Student's t-test. Results were considered statistically significant when* P*<0.05. All statistical analysis was performed using the statistical package SPSS13.0 (SPSS Incorporated, Chicago).

## 3. Results

### 3.1. Histological Analysis

**Figure 1:**
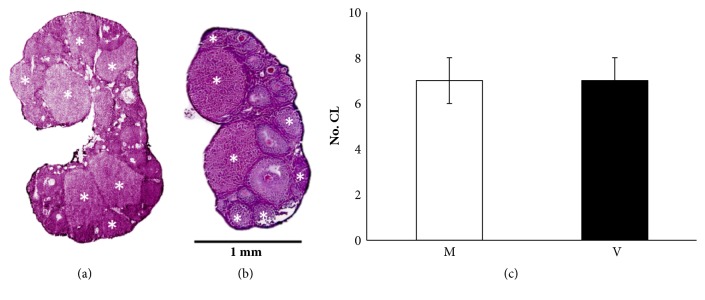
Quantification of corpora lutea number (CL) by H&E staining. Mother: M; virgin: V. In the representative images of ovaries from M (a) and V (b) mice, CLs were indicated by (*∗*). Data from 4 different experiments are expressed as the mean ± SEM (c). Bar= 1 mm.

The analysis of ovarian morphology was performed to confirm that both parous (Mothers, M) and nulliparous (Virgins, V) mice were at postovulatory stage of estrus cycle [27]. The ovaries of both M and V mice showed similar morphological characteristics and number of corpora lutea (CL) (Figure 1; M versus V,* P*>0.05).

### 3.2. qRT-PCR

**Figure 2:**
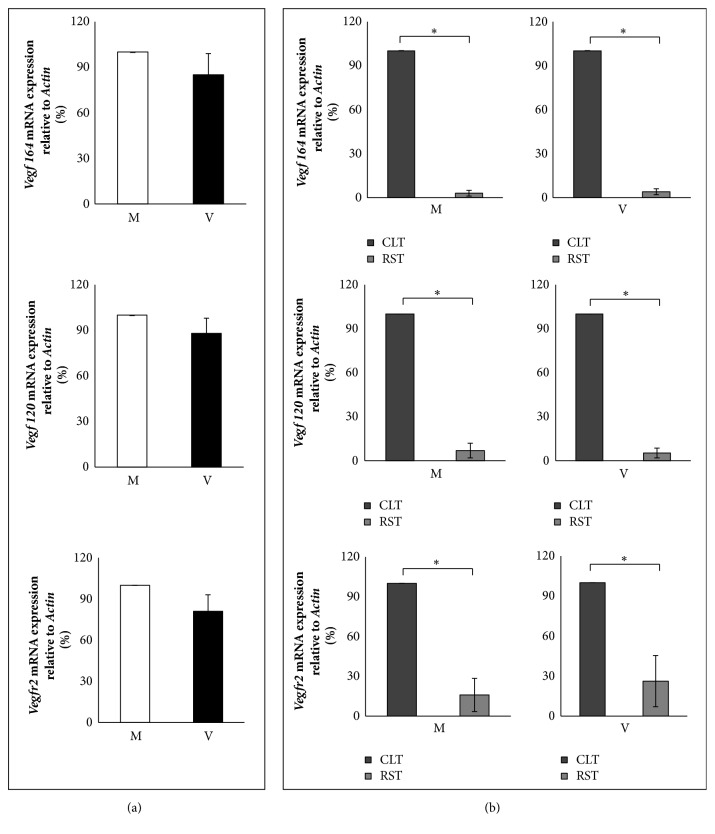
mRNA expression of Vegf 164 (upper panel), Vegf 120 (middle panel), and Vegfr2 (lower panel) relative to Actin. Mother: M; virgin: V; corpus luteum tissue: CLT; residual stromal tissue: RST. Similar Vegf 164, Vegf 120, and Vegfr2 mRNA levels are present in whole ovaries of M and V mice (a), while a different distribution occurs between CLT and RST (b). Bar graph data represent the mean percentage ± SEM of 3 independent experiments. Relative quantitative evaluation of mRNAs was performed by the comparative ΔΔCt method. Data from V mice are compared to data obtained from M mice, which are arbitrarily represented as 100%. Data from RST are compared to CLT, which are arbitrarily represented as 100%. *∗*P<0.05.

Analysis of* Vegfa 164* and* 120* isoforms of* Vegfr2* transcripts were reported in Figure 2. In whole ovaries, mRNAs were expressed similarly between M and V mice (Figure 2(a);* P*>0.05). Even though* Vegf 164 *mRNA levels were slightly lower, there was no statistical difference in comparison with* Vegf 120 *levels (*P*>0.05). Concerning ovarian mRNA distribution, results show that both* Vegfa* isoforms and* Vegfr2* mRNAs were preferentially accumulated in corpus luteum tissues (CLT) rather than residual stromal tissues (RST) (Figure 2(b);* P*<0.05).

### 3.3. Western Blot Analysis

**Figure 3:**
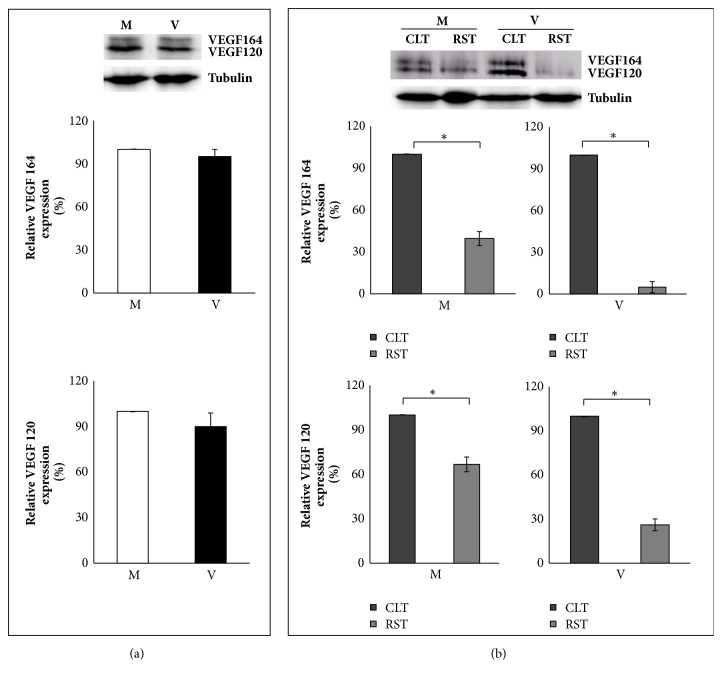
Representative images and protein quantification of VEGF 164 and VEGF 120. Mother: M; virgin: V; corpus luteum tissue: CLT; residual stromal tissue: RST. Levels of VEGF 164 (upper and middle panel) and VEGF 120 (upper and lower panel) in whole ovaries of M and V mice (a) and their distribution in CLT and RST (b). As shown by the representative images (a, upper panel), ovarian VEGF 120 content is almost 2-fold more than VEGFA 164 (P<0.05). Bar graph data represent the mean percentage ± SEM of 4 independent determinations. Data from V mice are compared to data obtained from M mice, which are arbitrarily represented as 100%. Data from RST are compared to CLT, which are arbitrarily represented as 100%. Comparisons are made within the same blot and across different blots. *∗*P<0.05.

In whole ovaries, levels of VEGFA 120 and 164 isoforms were comparable between V and M mice (Figure 3(a);* P*>0.05), with a clear predominant expression of isoform 120 (almost 2-fold more than 164;* P<*0.05). As reported in Figure 3(b), both VEGFA 120 and 164 were more abundantly expressed in CLT than RST (Figure 3(b);* P*<0.05).

**Figure 4:**
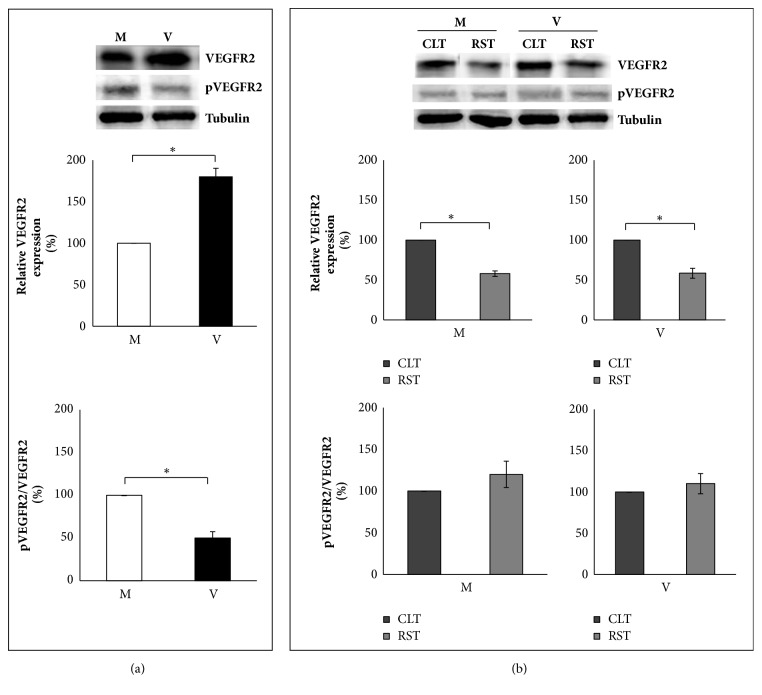
Representative images and protein quantification of VEGFR2 and pVEGFR2 (Y1173). Mother: M; virgin: V; corpus luteum tissue: CLT; residual stromal tissue: RST. The figure shows the different levels in whole ovaries of M and V mice (a) of VEGFR2 (upper and middle panel) and pVEGFR2/VEGFR2 ratio (upper and lower panel) and their distribution in CLT and RST (b). Bar graph data represent the mean percentage ± SEM of 4 independent determinations. Data from V mice are compared to data obtained from M mice, which are arbitrarily represented as 100%. Data from RST are compared to CLT, which are arbitrarily represented as 100%. Comparisons are made within the same blot and across different blots. *∗*P<0.05.

VEGFR2 quantification disclosed that the receptor was more accumulated in ovaries of V than M mice (Figure 4(a), upper and middle panel;* P*<0.05) and more predominantly in CLT (Figure 4(b), upper and middle panel; CLT versus RST,* P*<0.05). With respect to 1173-Tyr phosphorylation, the ratio pVEGFR2/VEGFR2 recorded in V ovaries decreased significantly compared to M (Figure 4(a), upper and lower panel;* P*<0.05). Nevertheless, in both groups the phospho-protein appeared equally distributed in the somatic compartments (Figure 4(b), upper and lower panel; CLT versus RST,* P*>0.05).

Moreover, total and phosphorylated forms of PLC*γ*1 and ERK1/2, both VEGFR2-activated downstream signals, were analyzed by western blot. Quantification of the aforementioned proteins evidenced very low and comparable expression levels between M and V mice (*P>*0.05, data not shown).

## 4. Discussion

This study demonstrates that VEGFR2 protein and phospho-protein levels are differently modulated in ovaries collected from primiparous (M) and nulliparous (V) mice.

Indeed, any parity-dependent effects on* Vegfa* (either* 120* or* 164*) and* Vegfr2* gene expressions were excluded because of comparable mRNA levels detected in the ovaries of both groups. Moreover, in agreement with literature data obtained on virgin animals [32, 33], also in M mice the expression of* Vegf* and* Vegfr2* mRNAs occurs predominantly in CLT. Our results confirm that in ovaries of mice and large mammals, the predominant* Vegf* isoforms are 120 and 164 [29, 30], whereas proangiogenic isoform 144 is expressed at very low level and 188 is completely absent in mice [29].

At protein level, our results show that even if ovarian VEGFA 120 protein is more expressed than the 164 form, their respective contents in the ovaries of M and V mice are comparable. Both proteins are preferentially present in corpus luteum tissues (CLT), thus mirroring mRNAs distribution. We do not know why ovarian VEGFA 120 and 164 protein contents differ, nor which of the two isoforms has a prominent role in our mouse model. As a matter of fact, also in other tissues and organs the basic mechanisms by which the different VEGFA isoforms operate are yet to be defined [34].

Although VEGFA 120 and 164 contents are significantly higher in corpus luteum tissue (CLT) of V mice in comparison with M mice, both VEGF isoforms are much more expressed in the residual stromal tissues (RST) of M than V mice. These findings suggest that pregnancy could more efficiently regulate follicle maturation, as already demonstrated in non-human primates and rodents [35, 36].

According to available human and rodent data [13, 37], also in our experiments VEGFR2 has been detected mainly in CLT, thus supporting the hypothesis that some cells of granulosa-lutein tissue could act as an endothelial-like cell population [13]. Despite similar mRNA expression, we found that ovarian VEGFR2 protein is more accumulated in V than M mice. This indicates that a parity/nulliparity-dependent regulation of receptor stability/degradation could occur. Literature data show that the dysregulation of the synthesis/degradation of other pro/antiangiogenic factors, and/or of the transcription factor hypoxia inducible factor-1 alpha (HIF-1*α*) could affect the expression and activation of this receptor [9, 19]. In our model, a different reactivity to hypoxic stress has been ruled out because of similar levels of* Vegf/Vegfr2* mRNA (present results) and of HIF-1*α* protein in both M and V mice (our unpublished data). Unfortunately, many of the events are involved in VEGFR endocytosis/trafficking and ubiquitylation, and also details of signaling pathways are not completely understood, and further studies are necessary to clarify all these issues [38]. However, it is generally accepted that low VEGFR2 levels are normally detected in adult vasculature and that the expression of this receptor is upregulated during inflammation or tumor growth [39].

The assessment of phosphorylation of VEGFR2 at Tyr 1173, that is, the major proangiogenic signal for the induction of endothelial cell proliferation [9], reveals that only a fraction (~50%) of total receptor is phosphorylated in the ovaries of V mice in comparison with M mice, where almost all receptor (~90%) is phosphorylated. However, since V mice used in present experiments are healthy and young and therefore potentially fertile, it is not surprising that pVEGFR2 content and distribution in CLT and RST of V mice are similar to those recorded in M animals. From literature data, the overactivation of VEGFR2 represents a negative event in the generation of a pathological proangiogenic environment [40, 41]. Therefore, we can speculate that phosphorylation of all receptor molecules could have more dramatic effects in nulliparous than in parous mice, because of a potentially stronger proangiogenic stimulus.

Among the various phosphotyrosine residues, it seems noteworthy that phosphorylation of Tyr1173 stimulates the activation of phospholipase C*γ*- (PLC*γ*-) dependent proangiogenic ERK pathways [38]. When we analyzed these proteins in our samples, we found low signals in both M and V ovaries, thereby supporting the hypothesis that VEGFR2 signaling is not altered in the ovaries of M and V mice and that a yet unknown mechanism prevents complete VEGFR2 phosphorylation in V ovaries.

## 5. Conclusion

In conclusion, we suggest that nulliparity and parity differently modulate ovarian VEGFA/VEGFR2 system and that more favorable conditions to potentially altered ovarian angiogenesis more likely occur in nulliparous than in parous females. Our results could, at least in part, help to explain epidemiological data showing that OC incidence is significantly higher in infertile women and especially in nuns [25].

## Figures and Tables

**Table 1:** Primer sequences for RT-PCR. Note: R1: reverse; F1: forward.

Oligo name	Gene target	Sequence (5′-3′)
mbeta actin R1	*Actin*	5′-TGGACAGTGAGGCCAGGATG-3′
mbeta actin F1	*Actin*	5′-TCGTGCGTGACATCAAAGAG-3′
mFlk1 R1	*Vegfr2*	5′-GACAGAGGCGATGAATGGTG-3′
mFlk1 F1	*Vegfr2*	5′-GAGAGCAAGGCGCTGCTAGC-3′
mVEGF 120 R1	*Vegf 120*	5′-CGGCTTGTCACATTTTTCTGGC-3′
mVEGF 120 F1	*Vegf 120*	5′-GAAGTCCCATGAAGTGATCAAG-3′
mVEGF 164 R1	*Vegf 164*	5′-CAAGGCTCACAGTGATTTTCTGGC-3′
mVEGF 164 F1	*Vegf 164*	5′-GAAGTCCCATGAAGTGATCAAG-3′

## Data Availability

Molecular data used to support the findings of this study are included within the article and available from the corresponding author upon request.
